# Aminoguanidines: New leads for treatment of *Giardia duodenalis* infection

**DOI:** 10.1016/j.ijpddr.2019.04.003

**Published:** 2019-04-08

**Authors:** Rebecca J. Abraham, Sam Abraham, Andrew J. Stevens, Stephen W. Page, Adam McCluskey, Darren J. Trott, Ryan M. O'Handley

**Affiliations:** a The University of Adelaide, School of Animal and Veterinary Science, Roseworthy, South Australia, 5352, Australia; b Murdoch University, School of Veterinary and Life Science, Murdoch, Western Australia, 6150, Australia; c Chemistry, School of Environmental and Life Sciences, The University of Newcastle, University Drive, Callaghan, NSW, 2308, Australia; d Neoculi Pty Ltd, Burwood, New South Wales, Australia

**Keywords:** *Giardia duodenalis*, Giardiasis, Drug development, Antigiardial, Giardicidal, Adherence

## Abstract

*Giardia duodenalis* is an ubiquitous parasitic pathogen that causes significant morbidity and mortality worldwide. Failures in drug therapy are commonly due to poor patient compliance as a result of the need for repeated administration, off target drug effects and increasing parasite drug resistance. In this study the *in vitro* efficacy and selectivity of the aminoguanidine compound robenidine and 2 structural analogues against *Giardia* were determined. After 5 h exposure to each compound the IC_50_ was as low as 0.2 μM with corresponding MLCs as low as 2.8 μM. This is in contrast to metronidazole which required 24 h to exhibit inhibitory activity. A modified adherence assay, developed for this study, demonstrated that three of the compounds inhibited *in vitro* adherence of the parasite. The lead compound exhibited rapid giardicidal activity (<5hr). In addition, microscopy studies demonstrated damage to the plasma membrane of trophozoites. In conclusion, a class of aminoguanidines, represented by robenidine, has shown antigiardial activity warranting further investigation.

## Highlights

• Robenidine has faster antigiardial activity *in vitro* compared to metronidazole.
• Novel structures developed with improved antigiardial activity compared to robenidine.
• Giardia is unable to recover after short exposure times to novel antigiardials
• Electron microscopy images show detailed structural damage after robenidine exposure.

## Graphical abstract

**Figure:**
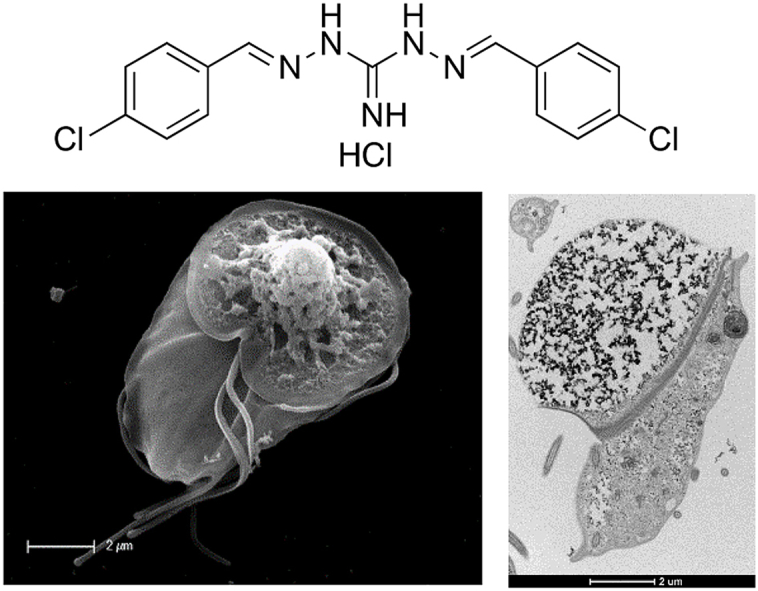

## 1 Introduction

*Giardia duodenalis* (syn. *Giardia lamblia, Giardia intestinalis)* is a bi-nucleate protozoan pathogen estimated to cause between 130 million and 262 million human infections annually (Kirk et al., 2015), making it the most common protozoal pathogen worldwide (Halliez and Buret, 2013; Upcroft and Upcroft, 1998, 2001). Although infections are common in developed countries they are more prevalent in developing nations. Giardiasis has been recognised by the World Health Organisation as a neglected disease causing widespread morbidity worldwide (Savioli et al., 2006). *Giardia* infection is acquired via ingestion of cysts, either directly through a faecal-oral route or by contaminated food or water (Savioli et al., 2006; Thompson, 2000). *Giardia* results in a malabsorptive gastrointestinal disease with symptoms including diarrhoea, bloating and abdominal cramping (Buret, 2008). Symptoms may be acute or chronic and re-occurring. Persistent infection, especially in children and immunocompromised hosts, results in long term effects including malnutrition, developmental delay and failure to thrive syndrome (Wright et al., 2003).

Current antigiardial drugs used to treat giardiasis are drawn from the nitroimidazole, nitrothiazole, nitrofuran, acridine, benzimidazole, quinolone and aminoglycoside structural classes (Wright et al., 2003). The most frequently used nitroimidazoles, metronidazole and tinidazole, have a treatment success rate of 80–90%; while albendazole, a benzimidazole, has a reported efficacy of 62–95% (Wright et al., 2003). Treatment failures with these drugs are frequently reported and many exhibit unwanted side effects including but not limited to, nausea, fatigue and malaise (Wright et al., 2003). Metronidazole, is known to cause vomiting, weakness and headaches and is potentially carcinogenic (Nagel and Aronoff, 2015; Bendesky et al., 2002; Jokipii and Jokipii, 1979). Furthermore, treatment failure due to the development of resistant organisms has been reported for all commonly used antigiardial drugs (Nagel and Aronoff, 2015; Jokipii and Jokipii, 1979).

**Fig. 1:**
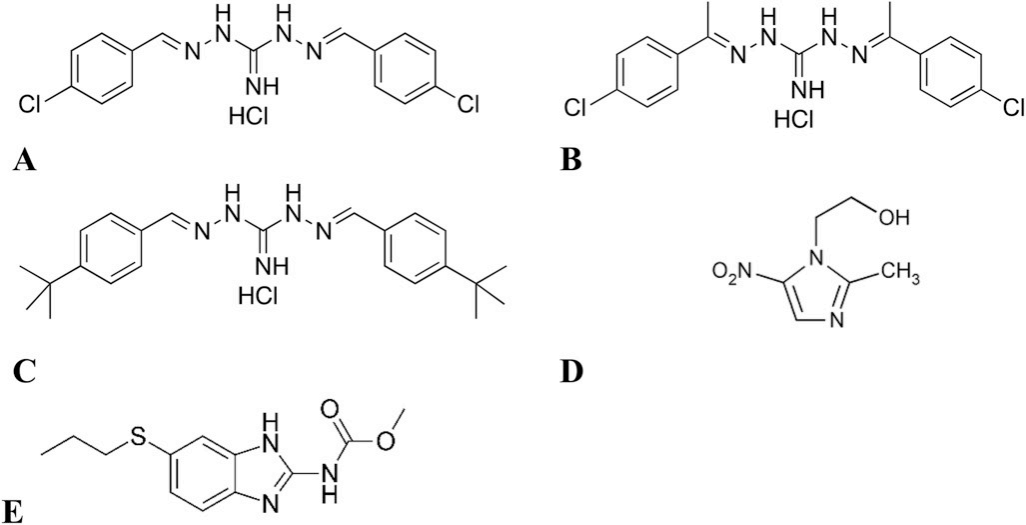
Chemical structures of the four compounds investigated in this study. **A**. robenidine; **B**. NCL 062; **C**. NCL 099; **D.** Metronidazole; and **E.** Albendazole.

The combination of ineffective treatments resulting from adverse side effects and emerging resistance to all classes of antigiardial drugs provides an imperative to identify and develop low side effect, low toxicity antigiardial compounds. In this study we explored the potential of robenidine, a symmetrical chloroaromatic compound linked via a guanidinal core, as a lead compound for the development of novel antigiardial drugs (Fig. 1). Robenidine has been in use in the commercial poultry and rabbit industries as an anticoccidial agent since the early 1970s (Kantor et al., 1970). As part of an on-going antigiardial drug development program, in-house screening identified robenidine as possessing antigiardial effects, but with undesired off target actions (Abraham et al., 2016). We thus sought to more closely examine exemplar guanidinal linked aromatic compounds as potential antigiardial agents with an improved safety profile. Herein, we evaluated the antigiardial activity of robenidine and the activity of two structural analogues, (*E*)-*N*'-((*E*)-1-(4-chlorophenyl)ethylidene)-2-(1-(4-chlorophenyl)ethylidene)hydrazine-1-carboximidhydrazide hydrochloride (NCL 062) and *N*′,2-bis((*E*)-4-(*tert*-butyl)benzylidene)hydrazine-1-carboximidhydrazide hydrochloride (NCL 099) (Fig. 1).

## 2 Materials and methods

### 2.1 Chemicals

Robenidine was provided by Neoculi Pty Ltd (Burwood, Vic, Australia). NCL 062 and NCL 099 were synthesised at the University of Newcastle (Abraham et al., 2016). The remaining drugs used in this study were sourced from Sigma Chemical Company (St Louis, Missouri).

Chemicals used for the culture of *Giardia in vitro* were sourced as follows: glucose and L-cysteine (ACROS organics, Thermo Fisher Scientific, Scoresby, Vic), ammonium iron (III) citrate, ascorbic acid (Sigma-Aldrich, Castle Hill, NSW), potassium dihydrogen orthophosphate (UNIVAR, Ingleburn, NSW), bovine bile (Fluka analytical (BD)), di-potassium hydrogen orthophosphate (Fronine Laboratories and Supplies, Riverstone, NSW).

### 2.2 Cell culture

*G. duodenalis (*BAH2c2 and BAH12 strain) was cultivated according to the method of Clark and Diamond in Keister's modified TYI-S-33 medium, supplemented with heat inactivated foetal bovine serum (Hyclone™, Thermo Fisher Scientific, Scoresby, Victoria, Australia) at 37 °C in plastic 9 mL screw-capped test tubes (nunc, Thermo Fisher Scientific, Scoresby, Victoria, Australia). Subcultures were performed once a confluent monolayer was observed, approximately 2–3 times per week (Clark and Diamond, 2002).

### *2.3* *In vitro* drug efficacy assays

#### 2.3.1 Resazurin reduction assay

The *in-vitro* compound efficacy was determined using a resazurin reduction assay as previously described (Benere et al., 2007). The media of confluent cultures was replaced with fresh media and the cultures were placed on ice for 40 min to detach trophozoites. Trophozoites were enumerated using a haemocytometer and 50 000 trophozoites were added to each test well of a 96 well plate. Doubling or tripling dilutions of the test compounds were added to wells beginning at 25 or 50 μM compound concentration (previously prepared in DMSO). Metronidazole and DMSO (vehicle only) were used as controls. Plates were incubated under anaerobic conditions, using anaerobic gas generating sachets, (AnaeroGen, Thermo Fisher Scientific, Scoresby, Victoria, Australia) for 24 h at 37 °C. After incubation the media was removed and replaced with an equal volume of warm PBS. Resazurin (Alamarblue, Thermo Fisher Scientific, Scoresby, Victoria, Australia) was then added at 10% of the total volume of the wells. Plates were further incubated (1.5 h) for colour development and absorbance read at 570 nm and 630 nm. The percentage resazurin reduction was then calculated using the following formula:

((ε_oxi_630 x A_570_) – (ε_oxi_570 x A_630_))/((εred_570_ x C_630_)-(ε_red_ x C_570_)) x 100

Where: ε_oxi_630 = 34798, ε_oxi_570 = 80586, A_570_ = absorbance at 570 nm, A_630_ = absorbance at 630 nm, ε_red_570 = 155677, ε_red_630 = 5494, C_630_ = absorbance of negative control well at 630 nm and C_570_ = absorbance of negative control well at 570 nm.

The effect of the compounds on the proliferation of *Giardia* trophozoites was determined following the protocol outlined above, with the following modifications. The number of trophozoites was decreased to 1000 per well and the cells were incubated in the presence of each compound for 96 h at 37 °C.

#### 2.3.2 Modified adherence assay

A second screening method, based on counting adherent trophozoites, was developed and used to validate the primary screen method, modified from earlier adherence assay protocols (Crouch et al., 1986). The assay was prepared in 24 well plates with plastic coverslips placed in the bottom. Trophozoites were prepared as above by placing on ice and ∼5 × 10^5^ cells/mL were added to each well. Drugs prepared in DMSO were added at the required concentration, with the DMSO concentration never exceeding 1%. Assay plates were incubated for 5 h at 37 °C under anaerobic conditions, the media removed and cells adhering to the coverslips were fixed with glutaraldehyde or methanol. Once the coverslips were dry, cells were stained with a Romanowsky stain variant (Rapid stain), fixed to a glass slide and imaged at 10x or 100x magnification. Images (10x magnification) were processed with DotCount v1.2 software (http://reuter.mit.edu/, 2008–2012 ^©^ Martin Reuter) to count the number of cells present and data analysed using GraphPad Prism version 6.00 for Windows, GraphPad Software, La Jolla California USA, www.graphpad.com. Images (100x magnification) were assessed for obvious morphological changes.

### 2.4 Mechanism of action

#### 2.4.1 Transmission electron microscopy

To determine any effects robenidine has on the ultrastructure of *Giardia* trophozoites, transmission electron microscopy (TEM) was performed. *Giardia* BAH2c2 trophozoites were exposed to 3 X IC_50_ of robenidine or metronidazole for 1 h and placed on ice for 40 min to detach trophozoites then washed twice with PBS (900×*g*, 10 min 4 °C) and fixed with a combination of glutaraldehyde and formaldehyde overnight. Cells were washed with PBS +4% sucrose before fixation with osmium tetroxide for 1 h. Samples were dehydrated through a graded ethanol series (70–100%) followed by suspension in propylene oxide for ten minutes. Samples were centrifuged and suspended in 1:1 mixture of propylene oxide and epoxy resin for an hour before centrifugation. Following overnight suspension in 100% epoxy resin samples were resuspended in fresh resin and polymerized at 70 °C for 24 h. After sectioning samples were stained with uranyl acetate and lead citrate. Sections were imaged with a FEI tecnai G2 Spirit Transmission Electron microscope (Adelaide Microscopy, University of Adelaide).

#### 2.4.2 Scanning electron microscopy

To determine the effect that robenidine and metronidazole has on the cell surface of the *Giardia* trophozoites, scanning electron microscopy (SEM) was performed as follows. *Giardia* BAH2c2 trophozoites were prepared as for TEM, however they were exposed to compounds for 2 or 4 h. After fixation with glutaraldehyde cells were attached to glass coverslips (pre-treated with poly-L-Lysine for 10 min) and washed with PBS + 4% sucrose before dehydration through a graded ethanol series (70–100%). Following ethanol dehydration coverslips were dried using a critical point drier, coated with platinum and observed using a ZIESS SEM (CMCA, University of Western Australia).

### *2.5* *Giardia* recovery assay

*G. duodenalis* trophozoites were harvested on ice as outlined above and 5 × 10^5^ cells/mL were added to 1.5 mL centrifuge tubes. Cells were exposed to 5x the IC_50_ of robenidine, metronidazole or DMSO (1%) only for 5 h under anaerobic conditions at 37 °C. After exposure cells were collected by placing tubes on ice for 40 min followed by centrifugation at 900×*g* for 5 min. The supernatant was removed and cells were resuspended in 8 mL of fresh media (in a 9 mL culture tube). The number of trophozoites was counted using a haemocytometer and cells were incubated for 48 h with cell numbers being determined at 24 and 48 h.

### 2.6 *In vitro* cytotoxicity

Human cells lines, Hep G2 (ATCC HB-8065) and HEL 299 (ATCC CCL-137), were maintained in Dulbecco's modified Eagle's medium (DMEM; Gibco cat no. 12430) supplemented with 10% foetal bovine serum and 1% PenStrep (100U/ml penicillin and 100 μg/mL streptomycin). Cells were passaged every 3 days. Assays were performed in 96 well plates in duplicate. Wells were seeded with 50 000 cells. Twenty four hours post seeding cells were washed and fresh media added. Two hours post wash, compounds diluted in DMSO were added in doubling dilution at a concentration of 1% DMSO. After 24 h exposure, WST-1 reagent at a final concentration of 10% was added to each well and absorbance at 450 nm was recorded after 1 h. The GI_50_ values were determined using the nonlinear regression function of GraphPad Prism v6 software.

### 2.7 Statistical analysis

The results for the *in vitro* drug efficacy studies were analysed using GraphPad Prism version 6.00 for Windows, GraphPad Software, La Jolla California USA, www.graphpad.com. For the resazurin reduction assay the mean and standard error of the mean were determined with each assay completed in triplicate. The IC_50_ was calculated using the log (inhibitor) vs. normalised response – variable slope function in Graphpad Prism. For the adherence assay mean and standard error of the mean were calculated and data analysed using an unpaired *t*-test, relative to untreated control.

## 3 Results and discussion

**Fig. 2:**
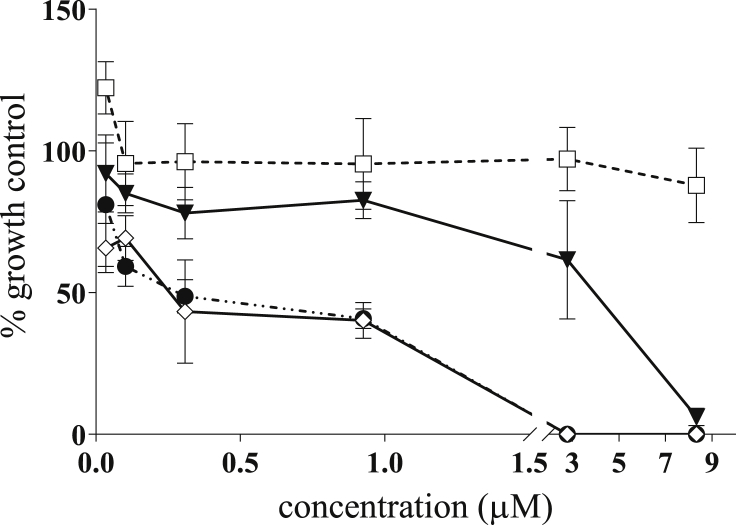
Metabolic inhibitory activity of robenidine, NCL 062 and NCL 099 against *Giardia duoenalis* BAH2c2 strain, assemblage A. Trophozoites were exposed to the compounds for 5 h before removal of the compounds and addition of resazurin. Absorbance was measured (630 and 570 nm), percent reduction of resazurin calculated and data presented as a percentage of the growth control. Treatment groups are: square - metronidazole; triangle –NCL 099; diamond - robenidine; and circle - NCL 062. Error ± SEM, n = 3. Data represents a typical experiment. Note the break in the x-axis indicates a change in the scale of drug concentration.

In this study, we demonstrated that robenidine, NCL 062 and NCL 099 have potent rapid *in vitro* activity against *G. duodenalis* (Fig. 2). The IC_50_ of each compound was determined using a resazurin reduction assay, which measures the metabolic activity of cells, using the log (inhibitor) vs. normalised response – variable slope function in Graphpad Prism. The compounds were tested against an assemblage A (BAH2c2, 24 h) and an assemblage B (BAH12, 24 h) isolate (Table 1). The parent molecule, robenidine, had antigiardial activity with an IC_50_ of 2.9 ± 2.9 μM and a minimum lethal concentration (MLC), the concentration at which no metabolism of resazurin was observed, of 2.8–8.3 μM after 24 h. This is similar to the current gold-standard treatment, metronidazole, which returned an IC_50_ of 2.0 ± 1.0 μM and an MLC of 2.8 μM. Both of the robenidine analogues tested displayed potent antigiardial efficacy with IC_50_ values of 3.0 ± 0.3 and 0.8 ± 0.6 μM respectively for NCL 099 and NCL 062. Variation in the MLCs was observed with NCL 062 having an MLC of 0.9 μM and NCL 099 with an MLC of 8.3 μM. Activity against the assemblage B isolate was similar to the results for the assemblage A isolate (Table 1). The effect of the compounds on the proliferation of *Giardia* trophozoites (BAH2c2) was also determined. The IC_50_ determined above and the IC_50_ of proliferation were similar for all compounds except NCL 099 where the IC_50_ of proliferation was 4-fold higher than the IC_50_. (Table 1).

**Table 1:** Antigiardial IC_50_ values and MLC for robenidine, NCL 099, NCL 062, metronidazole and albendazole against *Giardia duodenalis* assemblage A and B after 24 h. Data were obtained using a resazurin metabolic assay. IC_50_^prol^ refers to the concentration to cause 50% inhibition in the proliferation assay.

Compound	Assemblage A (μM)			Assemblage B (μM)		RAW264.7 (μM)
	IC_50_	IC_50_^prol^	MLC	IC_50_	MLC	GI_50_
Robenidine	2.9 ± 2.9	1.2 ± 0.7	2.8–8.3	4.7 ± 0.6	16.6	17.1
NCL099	3.0 ± 0.3	11.1 ± 0.8	8.3	3.3 ± 0.2	5.5	7.3
NCL062	0.8 ± 0.6	2.0 ± 1.3	0.9	1.8 ± 0.0008	3.1	4.0
Metronidazole	2.0 ± 1	1.7 ± 2.0	2.8	3.8 ± 0.5	16.6	NT
Albendazole	<0.02	0.8 ± 0.7	0.02–0.07	NT	NT	NT

**Fig. 3:**
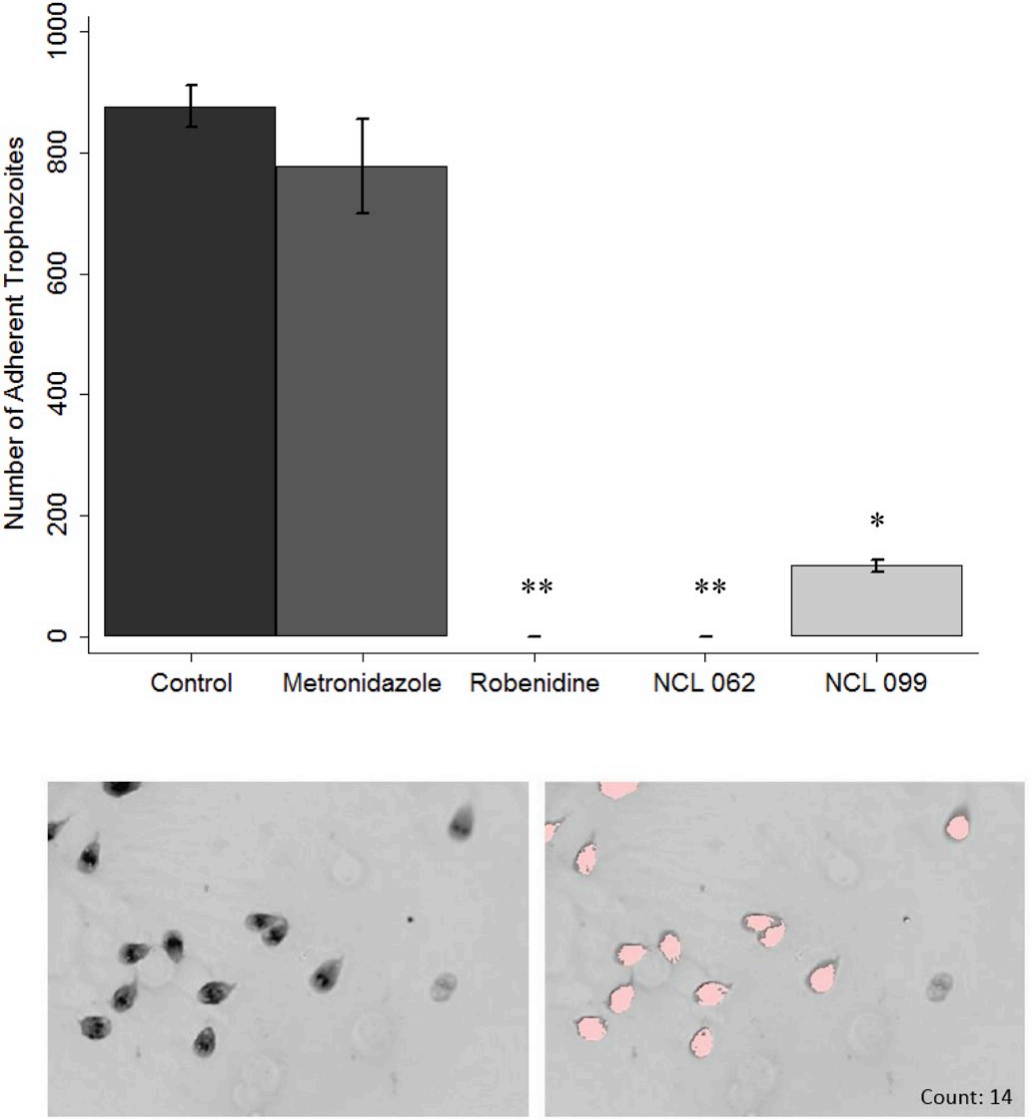
Inhibition of adherence of *Giardia duodenalis* by metronidazole (Mz), robenidine, NCL 062 and NCL 099. Cells were exposed to the compounds for 5 h before staining. Stained cells were imaged at 10x magnification and counted using DotCount™ software. Each assay was completed in triplicate and 5 images taken per sample. **Top.** Number of adherent trophozoites after exposure to 4 μM of each compound for 5 h; **Bottom.** An example of the Dotcount software identifying trophozoites at higher magnification. Left: stained trophozoites, Right: highlighted trophozoites identified by the Dotcount software. Each highlighted cell is counted individually. Error ± SEM. * *p*-value <0.05, ** *p*-value <0.0001.

**Fig. 4:**
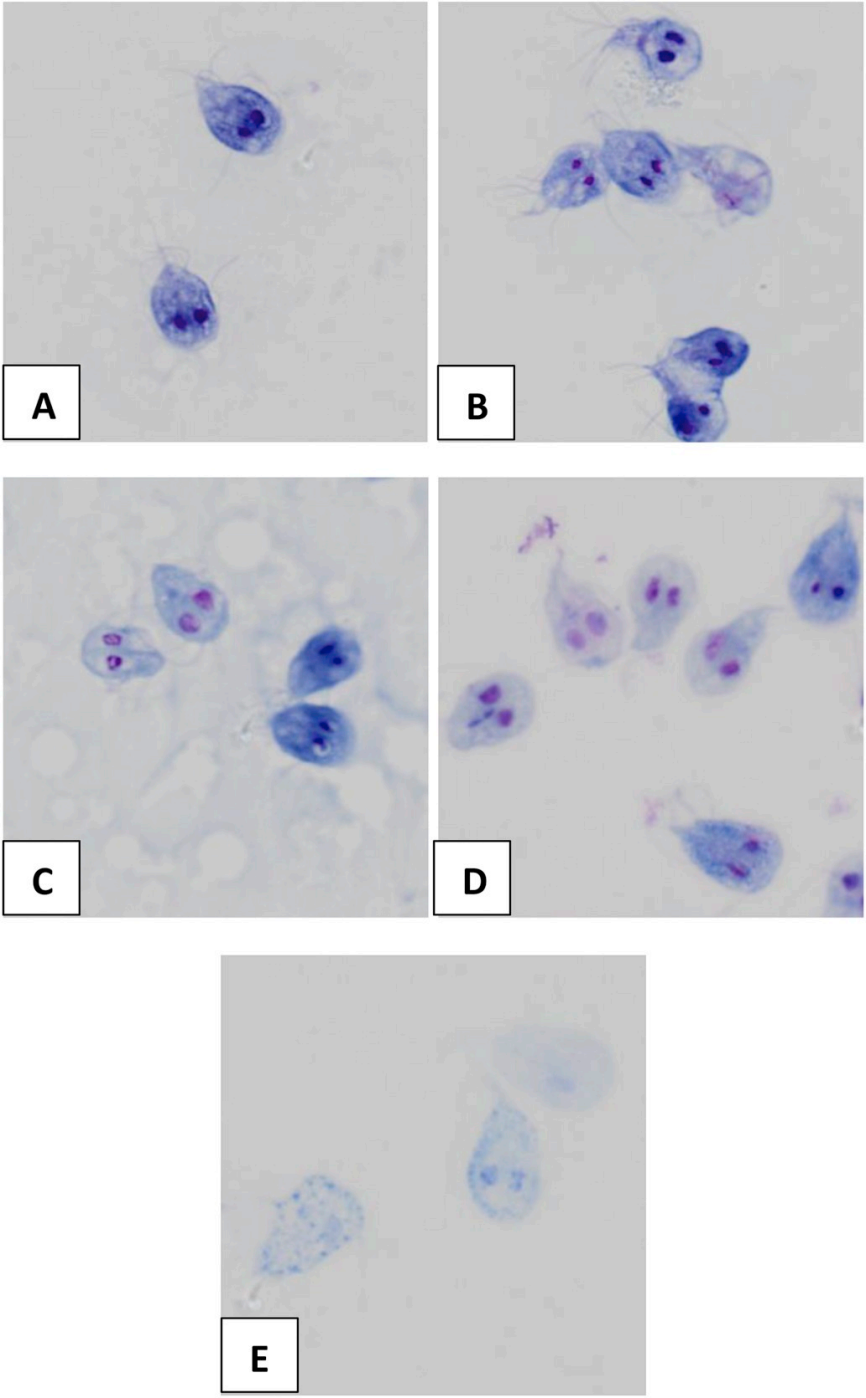
Morphological effect of Robenidine and NCL 099 on *Giardia* trophozoites. *Giardia* trophozoites were exposed to each compound for 24 h before fixation with glutaraldehyde and staining following the Diff quik protocol. A-control cells, B – cells treated with metronidazole (IC_50_, 2 μM), C- robendine treated cells (IC_50_, 2 μM), D – NCL 099 treated cells (IC_50_, 3 μM), E − NCL 099 treated cells (2 x IC_50_).

As trophozoite adherence is also a key characteristic in the establishment and development of *Giardia* infection, the effect of compounds on trophozoite adherence was examined further using a modified adherence assay. In contrast to metronidazole, robenidine, NCL 062 and NCL 099 significantly (p ≤ 0.05) affected the adherence of trophozoites at concentrations of 4 μM after 5 h exposure. With robenidine and NCL 062a complete inhibition of adherence was observed at 4 μM (Fig. 3). NCL 099 significantly affected adherence of trophozoites at 4 μM, but did not completely inhibit adherence. The effect of NCL 099 on cell membrane integrity was also observed in stained images of the trophozoites taken at 100 x magnification (Fig. 4). At the IC_50_ of NCL 099 (3.0 μM) a decrease in the membrane stain intensity was observed. At 2 x the IC_50_ of NCL 099 a disintegration of the cell membrane was observed (Fig. 4E). A decrease in the intensity of the membrane stain after treatment with robenidine at the IC_50_ (2.9 μM) was also observed and affected cells appeared to have enlarged nuclei (Fig. 4C). Previously published work has also demonstrated the mechanism of action of robenidine in bacteria is linked to disruption of the cell membrane in susceptible species (Ogunniyi et al., 2017). No cells could be imaged at higher concentrations of robenidine and NCL 062. Metronidazole had no significant effect on cell adherence at the time or concentration tested in this experiment, which is to be expected based on its known mechanism of action (Fig. 3) (Nagel and Aronoff, 2015).

Despite robenidine being used for the past 40 years as an anticoccidial agent its mechanism of action against protozoa is not known. Previous studies of the mechanism of action of robenidine have been inconclusive with some suggesting that ATPase is the primary target while others have identified no obvious morphological effect in *E. tenella* on mitochondria or the nucleus, with chiefly the Golgi body and endoplasmic reticulum being affected (Lee and Millard, 1972; Wong et al., 1972). In the same study, swelling of the perinuclear space was noted, it was suggested this could be due to the overproduction of proteins potentially indicating an increase in metabolism causing the cells to self-destruct (Lee and Millard, 1972). In the present study, we undertook electron microscopy studies as a first step to elucidate a possible mechanism of action of robenidine against *Giardia*.

**Fig. 5:**
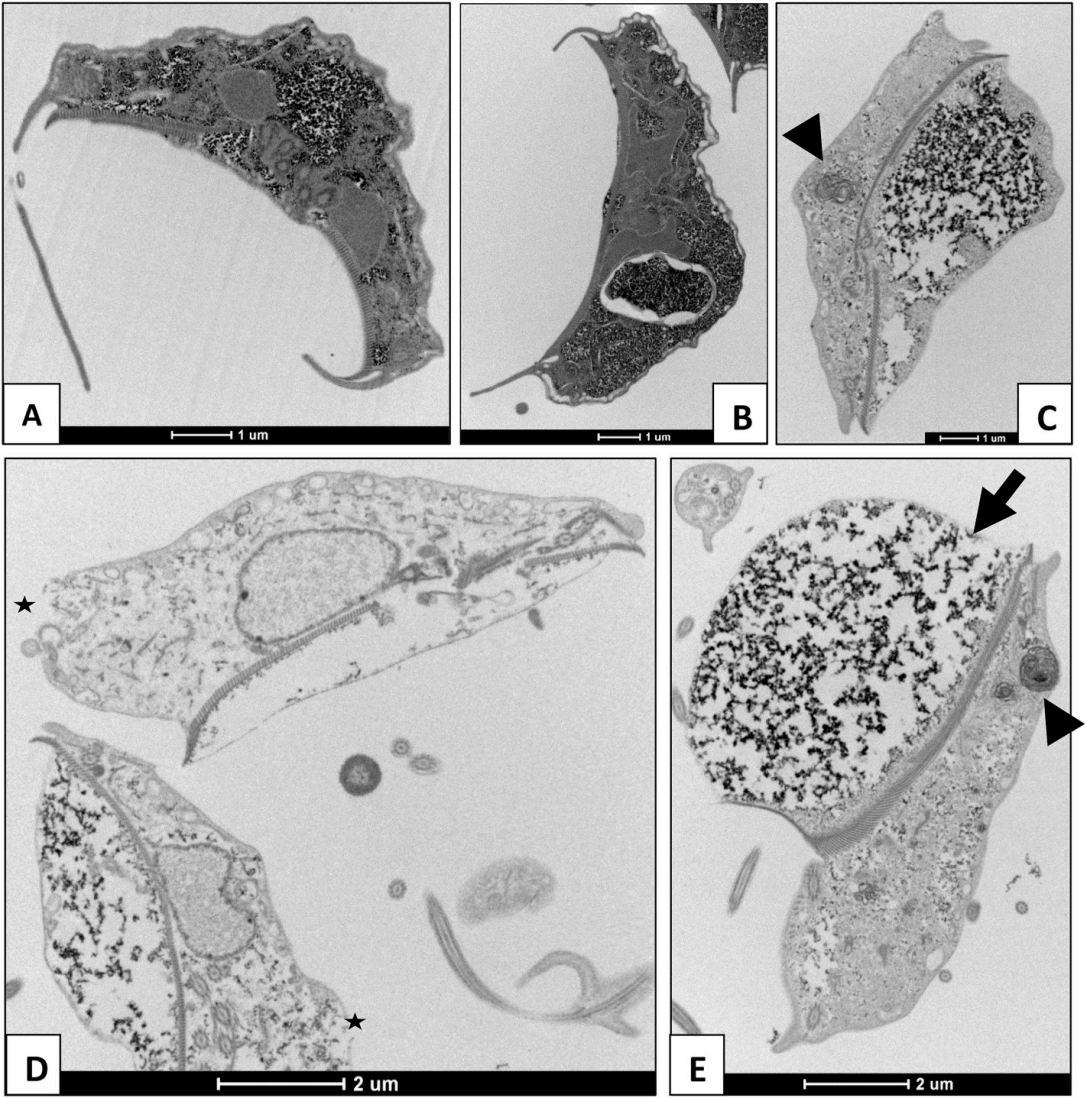
Transmission Electron Microscopy of *Giardia duodenalis* trophozoites: **A** control, and after exposure to **B** metronidazole for 4 h (3 x IC_50_) or **C-E** robenidine for 1 h (3 x IC_50_). Membrane blebbing at the adhesive disc can be seen in robenidine treated cells (arrows) as well as membranous structures within the cytoplasm (arrow head), disintegration of the cytoplasmic space and rupturing of the cell membrane (star). Images taken with a FEI tecnai G2 Spirit Transmission Electron microscope.

**Fig. 6:**
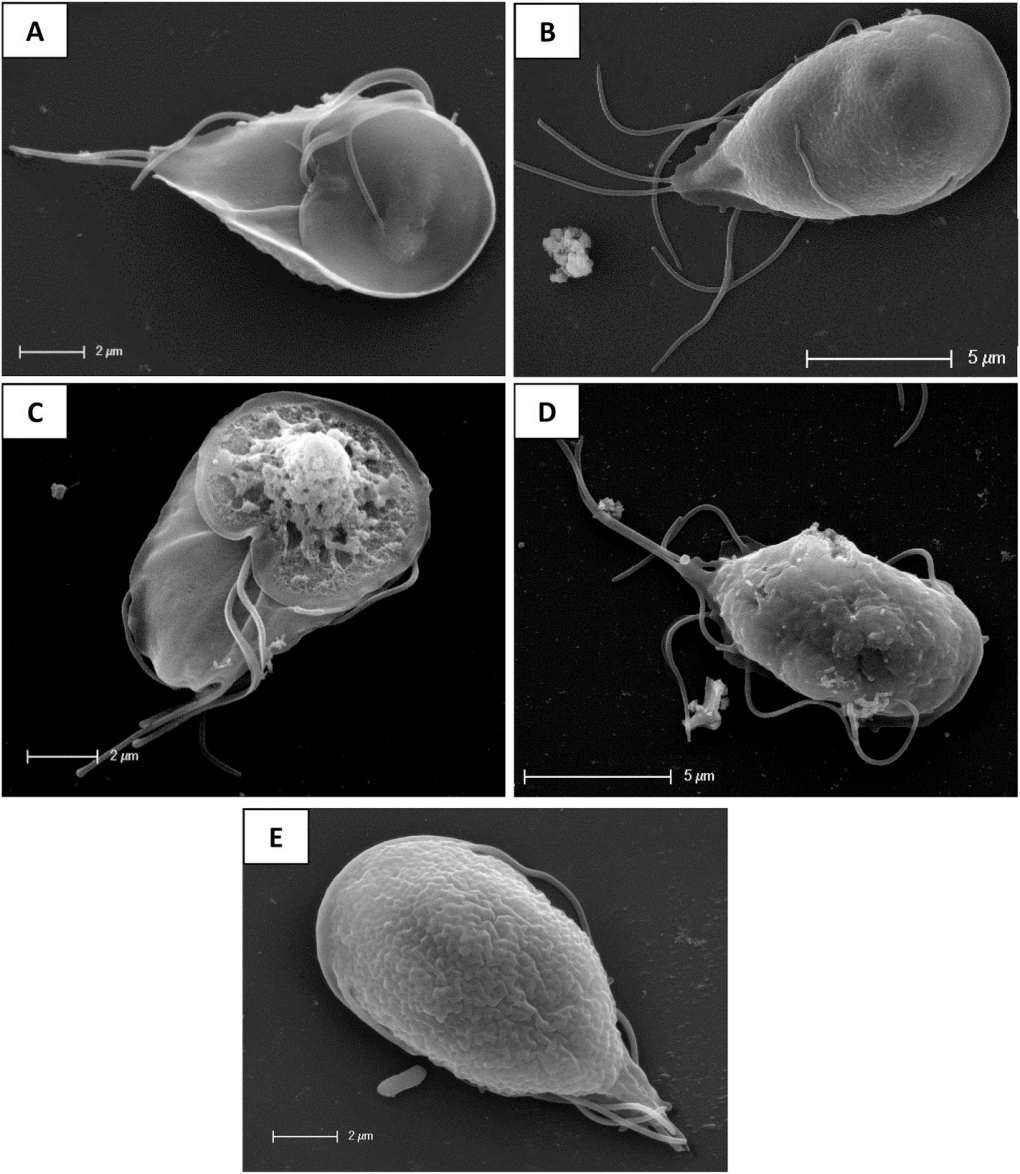
Scanning electron microscopy of *Giardia duodenalis* trophozoites: **A** control ventral, **B** control dorsal, **C** exposed to robenidine 2 h (3 x IC_50_) ventral, **D** exposed to robenidine 4 h (3 x IC_50_) dorsal. Multiple ruptures as well as blebbing at the adhesive disk can be seen on the ventral surface (C) while tiny blebbing and rupturing of the dorsal surface can be seen in D. **E** metronidazole exposed cells (3 x IC_50_) 4 h, dorsal.

Electron microscopy showed gross morphological changes in the trophozoites after 1 h of exposure to robenidine (Fig. 5). TEM studies revealed that the treated trophozoites developed extreme membrane blebbing, most significantly affecting the adhesive disc, scattered with electron dense material. In addition, unusual vacuolar membranous structures appeared within the cytoplasm. Several trophozoites also exhibited rupturing of the dorsal cytoplasmic membrane and all observed trophozoites had various degrees of disintegration of the cytoplasmic space. SEM studies also showed extreme membrane blebbing, as seen in TEM, after 2 h of exposure. Furthermore, these images showed that there was severe damage to the cell wall of the engorged adhesive disk with distinct lesions observed on the surface. Based on the electron microscopy results of this study it appears that robenidine exposure results in a general swelling of the cells leading to rupture of the cell membrane and ultimately cell death (Fig. 6).

It is possible that robenidine causes the plasma membrane of the protozoan to become destabilized, altering membrane properties leading to cell swelling and modifications of the cytoplasmic space. Another possible theory is that robenidine has a similar mechanism of action as the thiazolides which demonstrate a disintegration of the cytoplasmic space as observed by TEM and development of membrane ruptures on the adhesive disc resulting in a loss of osmotic potential (Muller et al., 2006). In addition, research into the mechanism of action of CGP 40215A, effectively a structural analogue of robenidine where the chlorides have been substituted with amine groups, identified a strong bond with the AT region of DNA (Nguyen et al., 2002). The strong bond was facilitated by the guanidine backbone which is conserved in robenidine, potentially providing another alternative mechanism of action for this series of compounds. The binding of robenidine analogues with appropriate structural, spatial and hydrogen bond characteristics to the AT region of DNA could result in a cascade of events resulting in a disruption of normal cellular process and eventually, cell death.

**Fig. 7:**
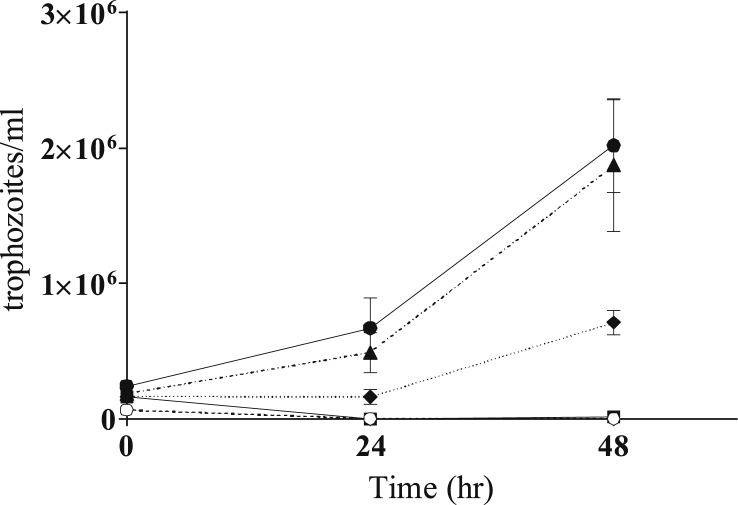
Recovery assay of *Giardia duodenalis* exposed to robenidine and metronidazole. *Giardia* trophozoites were exposed to robenidine or metronidazole for 5 h at 5x the IC_50_. Key: closed circle – growth control, triangle – metronidazole, square – robenidine, open circle – NCL 062, diamond – NCL 099. Cell numbers were determined at 24 and 48 h post exposure. Error ± SD.

Trophozoites exposed to robenidine and NCL 062 were unable to recover after short-term exposure (5 h) at 5x the IC_50_, again, in contrast to metronidazole which did not have any permanent effects after short exposure times, most likely due to the mechanism of metronidazole which requires metabolism to form nitro radicals (Upcroft and Upcroft, 2001) (Fig. 7). NCL 099 treated trophozoites were able to recover after short-term exposure but growth was slower when compared to untreated and metronidazole treated cells.

In addition to the potent *in vitro* and giardicidal activity, all three of the aminoguanidines studied here have the advantage of being highly insoluble in aqueous solution thereby potentially increasing the concentration at the site of infection by limiting systemic absorption from the gastrointestinal tract. While robenidine has been used extensively in the poultry and rabbit industries, we observed off-target antibacterial effects and cytotoxicity towards human lung fibroblast (HEL 299; GI_50_ = 17.7 μM) and human hepatocellular carcinoma (Hep G2; GI_50_ = 25.5 μM) cell lines. Cytotoxicity against murine macrophage cells for robenidine and both analogues was also observed (RAW 264.7; GI_50_ robenidine = 17.1 ± 4.4, NCL 062 = 4.0 ± 1.3, NCL 099 7.3 ± 2.8).

In conclusion, the results presented in this study demonstrated that the class of aminoguanidines related to robenidine, have potential to be developed as antigiardial agents. The three analogues presented here were potent, quick acting and have the potential to concentrate at the target site due to aqueous insolubility. These compounds provide a necessary starting point in the search for analogues with greater selectivity for *Giardia* and less off target effects against both host cells and the autochthonous bacteria of the gastrointestinal tract.
